# Emotional Modulation of Working Memory in Older Adults: Dissociable Contributions of Cognitive Reserve and Emotional Intelligence

**DOI:** 10.3390/brainsci16070725

**Published:** 2026-07-08

**Authors:** Stefania Lucia, Bianca Monachesi, Elisabetta Pisanu, Silvia Fornaro, Raffaella Ida Rumiati

**Affiliations:** Neuroscience Area, Scuola Internazionale Superiore di Studi Avanzati (SISSA), 34100 Trieste, Italy; slucia@sissa.it (S.L.); bmonache@sissa.it (B.M.); episanu@sissa.it (E.P.); sfornaro@sissa.it (S.F.)

**Keywords:** working memory, n-back, ageing, emotional intelligence, cognitive reserve, emotion–cognition interaction, older adults

## Abstract

**Highlights:**

**What are the main findings?**

- Cognitive reserve was positively associated with overall accuracy in an emotional n-back task across cognitive load and emotional content, supporting a domain-general contribution to working memory in older adults.
- Emotional intelligence selectively modulated response dynamics, but not accuracy, as a function of task relevance and emotional valence, with opposite effects on reaction times to happy faces across the Age and Emo tasks.

**What are the implications of the main findings?**

- Cognitive reserve and emotional intelligence contribute to working memory in ageing through partially dissociable mechanisms: the former preserves accuracy in a domain-general manner, whereas the latter selectively modulates response speed in emotionally salient contexts.
- Integrative models of cognitive ageing should account for both cognitive and emotional individual differences, as successful functioning in later life depends not only on preserved processing capacity but also on the flexible regulation of emotionally salient information.

**Abstract:**

**Background/Objectives:** Working memory declines with age, particularly under conditions of high cognitive load. Emotional information can modulate performance depending on its task relevance and on individual cognitive and emotional resources. This study investigated the dissociable contributions of cognitive reserve (CR) and emotional intelligence (EI) to accuracy and response dynamics in an emotional n-back task in older adults. **Methods:** Forty-five healthy older adults completed an emotional n-back task under low (1-back) and high (2-back) cognitive load. Task relevance was manipulated by requiring judgments either on facial expressions (Emo task) or on age-related features (Age task). Accuracy and reaction times were analyzed using mixed-effects models, with CR and EI entered as continuous covariates. **Results:** Higher cognitive load was associated with reduced accuracy and slower reaction times. Emotional modulation of performance emerged primarily when emotional information was task-relevant. Happy facial expressions were associated with faster reaction times and higher accuracy than angry expressions, particularly under high cognitive load. CR was positively associated with overall accuracy across conditions, without interacting with task demands or emotional content. In contrast, EI did not predict accuracy but selectively modulated reaction times as a function of task relevance and emotional valence, with opposite effects observed across tasks for positive stimuli. **Conclusions:** These findings indicate that emotional modulation of working memory in older adults is strongly context-dependent. CR supported accuracy in a domain-general manner, whereas EI selectively modulated response speed in emotionally salient contexts without conferring a direct accuracy advantage. Cognitive and emotional resources thus contributed to distinct yet interacting components of working memory performance in ageing, suggesting that successful functioning in later life depends not only on preserved processing capacity but also on the flexible regulation of emotionally salient information.

## 1. Introduction

Working memory is a core component of executive functioning, supporting the temporary maintenance and manipulation of information required for goal-directed behavior [1]. Despite its limited capacity, typically estimated at approximately four items, working memory shows substantial interindividual variability [2] and is characterized by a high degree of flexibility, allowing individuals to adapt behavior to changing contextual demands [3]. This system plays a critical role in everyday decision-making, influencing the selection and implementation of actions [4].

Classical models of working memory, such as Baddeley’s multicomponent framework, posit the existence of domain-specific subsystems (the phonological loop and the visuospatial sketchpad) coordinated by a central executive [5]. More recent theoretical accounts have challenged the notion of a unitary executive, emphasizing instead dynamic control mechanisms. In particular, gating models propose that working memory performance depends on the ability to flexibly open the gate to update salient information and close it to protect maintained representations from interference [6]. Extending this view, contemporary models conceptualize working memory as the outcome of a balance between flexibility and interference control, with interference arising from both representational overlap and competitive neural processes [3].

The n-back task is a widely used behavioral paradigm in neuropsychology for assessing executive functions, particularly working memory [7]. Task demands can be systematically manipulated by varying the value of n: participants are required to evaluate whether the current stimulus matches the one presented n positions earlier, thereby engaging processes of maintenance, updating, and monitoring [1]. In the 1-back condition, performance relies primarily on the comparison with the immediately preceding stimulus, whereas the 2-back condition imposes higher cognitive demands by requiring the active maintenance of stimulus order in addition to stimulus identity. Accordingly, higher difficulty levels are typically associated with reduced accuracy and increased processing costs [1,7]. The n-back task has been implemented using a wide range of stimulus materials, including numbers, words, geometric shapes, and faces [8,9].

More recently, emotional variants of the n-back task have been introduced to investigate how affective information modulates working memory updating and monitoring processes [10]. In such paradigms, emotional expressions are either directly relevant to the task or constitute contextual information that must be ignored.

This line of research reflects a growing interest in emotion–cognition interactions, according to which emotional salience can either enhance or impair cognitive performance depending on its relevance to task goals [11]. Empirical evidence indicates that emotional stimuli may facilitate working memory performance when they are relevant to task demands (e.g., [12,13]), whereas they may interfere with performance when they are task-irrelevant and, as such, distracting (e.g., [14]). This apparent inconsistency is coherently accounted for by Pessoa’s [15] dual competition model, which posits that emotional and cognitive processes compete for shared neural resources: emotional information enhances performance when it is goal-relevant, but impairs performance when it diverts attentional resources away from the task.

Importantly, understanding emotion–cognition interactions becomes particularly relevant in the context of ageing, as normal ageing is associated with well-documented changes in working memory efficiency, processing speed, and cognitive control [16,17]. Age-related declines in working memory updating and monitoring processes may increase susceptibility to interference, making older adults especially sensitive to the modulatory effects of emotional information [18].

Within this framework, emotional stimuli may play a compensatory or disruptive role in ageing, depending on task demands and the relevance of affective content to current goals. Emotional salience might thus differentially influence working memory performance in older adults, particularly in tasks requiring continuous updating such as the n-back paradigm [19]. More specifically, emotional salience may either support or disrupt performance depending on its alignment with task goals and on the availability of cognitive control resources.

Previous evidence indicates that emotional valence differentially affects working memory performance in ageing. In particular, Berger and colleagues [9] reported that positive emotional expressions selectively facilitate response speed, an effect not observed for neutral or negative stimuli. Moreover, broader evidence shows that, in older adults, the facilitating effects of emotion are especially pronounced in low-demand conditions, such as the 1-back task. Under such circumstances, emotional information may be processed as salient and meaningful even when cognitive demands are minimal, reflecting an increased prioritization of affective content in ageing.

This pattern is consistent with the motivational account proposed by socioemotional selectivity theory, according to which older adults preferentially orient toward emotionally positive and meaningful information as a function of perceived time horizons [20,21]. Importantly, such positivity-consistent patterns do not require a direct comparison with younger adults to be theoretically meaningful: they can manifest as a relative processing advantage for positive over negative material within older samples, particularly when cognitive resources are available to support goal-directed emotion regulation.

This pattern also aligns with the well-documented positivity effect, according to which older adults preferentially attend to and process emotionally positive and meaningful information [20]. From a functional perspective, this bias may support adaptive behavior by promoting emotional well-being; however, it may also interact with cognitive demands in complex tasks, influencing performance depending on available resources.

Because working memory efficiency declines with age, affecting both processing speed and accuracy [1,6], emotional processing has been proposed as a potential compensatory resource capable of supporting performance in older adults. Nevertheless, the extent to which such compensation occurs is likely to depend on individual differences in cognitive resources accumulated across the lifespan.

In this context, cognitive reserve (CR) has been identified as a key moderator of age-related cognitive decline. CR refers to the set of experiential resources derived from education, occupational complexity, and engagement in cognitively stimulating activities, which enable individuals to cope more effectively with age-related or pathological brain changes [22,23]. These resources are thought to preserve performance through more efficient cognitive strategies or the recruitment of alternative neural networks. Within dynamic control models of working memory, CR may enhance the efficiency of gating and interference resolution mechanisms, particularly under high-load conditions. Substantial evidence indicates that higher CR is associated with better working memory and executive functioning in older adults, particularly under conditions of increased cognitive demand [24,25].

Beyond CR, individual differences in emotional intelligence (EI) may further contribute to variability in working memory performance in ageing, especially with emotional stimuli. EI refers to the perceived ability to perceive, understand, regulate, and utilize emotional information in oneself and others [26]. In the present study, EI was operationalized as a trait-level construct, capturing self-reported emotional competencies rather than maximal emotional performance [27]. This self-reported dimension has been associated with more efficient emotion regulation, reduced emotional interference, and better cognitive performance in emotionally demanding contexts [28]. Indeed, Gutiérrez-Cobo et al. [29] showed that higher EI predicts better working memory performance in the presence of emotional stimuli compared to neutral conditions. Rather than uniformly enhancing performance, EI may shape strategic responding in emotionally salient contexts, influencing response dynamics without necessarily improving accuracy. In older adults, EI has been proposed as a potential resource supporting adaptive emotion–cognition interactions, particularly in tasks involving emotional stimuli, by facilitating goal-directed processing and limiting distraction from task-irrelevant affective content [30]. However, the extent to which EI modulates working memory performance across different task demands and emotional contexts in ageing remains insufficiently explored.

Taken together, CR and EI can be conceptualized as two complementary, yet mechanistically distinct, resources that may support successful cognitive functioning in older adults. CR reflects a domain-general, experience-based capacity that is thought to operate independently of specific task content, buffering against age-related decline by promoting more efficient and flexible use of available neural and cognitive resources. EI, by contrast, is specifically related to the processing of emotionally salient information and may therefore exert its influence selectively in contexts in which affective content is task-relevant, shaping how such information is prioritized, regulated, and integrated into ongoing performance. Examining CR and EI within the same task and sample provides an opportunity to determine whether these two individual-difference dimensions support working memory through dissociable mechanisms: a general, context-independent contribution for CR versus a context-dependent, emotion-specific contribution of EI, rather than reflecting a single underlying construct.

Based on the theoretical framework outlined above, we tested the following hypotheses. First, we expected accuracy to be lower and reaction times to be slower under high cognitive load (2-back) than under low cognitive load (1-back), reflecting the well-established cost of increased working memory demand. Second, we expected emotional modulation of performance to emerge selectively when emotional content was task-relevant (Emo task) rather than task-irrelevant (Age task), consistent with the dual competition model, with happy expressions yielding facilitated performance relative to angry expressions. Third, we expected higher CR to be associated with better accuracy in a domain-general manner, independently of task type, emotional content, and cognitive load. Fourth, we expected higher EI to selectively modulate reaction times, rather than accuracy, as a function of task relevance and emotional valence, reflecting a context-dependent contribution of EI to emotion–cognition interactions in older adults.

## 2. Materials and Methods

### 2.1. Participants

Forty-five older adults (28 women) were recruited through advertisements posted in the city of Trieste, a volunteer database, and the local community (see Table 1 for descriptive statistics of socio-demographic and cognitive characteristics). Participants ranged in age from 65 to 84 years (*M* = 69.83, *SD* = 5.90, see Table 1). The data reported here are part of a broader research project involving the same participant pool, which also completed additional tasks and was compared with other samples (see [31]). The emotional n-back task, the variables, and the analyses presented in this study are novel and do not overlap with those reported in [31], which addressed different tasks and research questions.

An a priori power analysis was conducted using G*Power version 3.1 [32], assuming a conservative effect size (f = 0.40), a significance level of α = 0.05, and a power of 0.85. This effect size was selected to provide adequate statistical power even under more conservative assumptions than those reported in comparable previous studies employing emotional n-back paradigms with similar factorial designs in younger and older adult samples, which reported effect sizes ranging from small-to-medium to large for the experimental manipulations of interest [9,10]. The power analysis was calculated for the within-participants effects of the mixed-effects models reported in this study (Task, Emotion, Difficulty, and their interactions) and indicated that a minimum sample size of 11 participants was required to achieve the specified power. The final sample of 45 participants, therefore, exceeded this requirement, indicating that the study was adequately powered to detect the effects of interest. All statistical analyses were performed in RStudio 2026.06.0 using mixed-effects models. Inclusion criteria were: age ≥ 65 years; normal or corrected-to-normal vision; right-handedness; and a Montreal Cognitive Assessment (MoCA) score at or above the dementia cutoff (≥17), confirming the absence of clinically significant cognitive impairment. Exclusion criteria were: a documented history of neurological or major psychiatric disorders; uncorrected sensory impairments (visual or auditory) that could interfere with task performance; current use of psychoactive medication known to affect cognitive or emotional processing (e.g., benzodiazepines, antidepressants with marked sedative effects); and any medical condition that, in the judgment of the research team, could compromise the participant’s ability to complete the experimental tasks safely or reliably. Written informed consent was obtained from all participants, and they received €20 compensation at the end of the experiment. The study was approved by the SISSA Ethics Committee (Prot. n. 6858-III/13, 20 February 2024).

### 2.2. Materials and Procedure

#### 2.2.1. Questionnaires

The study consisted of two experimental sessions. In the first experimental session, all participants completed a neuropsychological assessment, the CR questionnaire, and Emotion Intelligence Scale (EIS). During the second session, participants completed the working memory n-back task.

For the general neuropsychological screening of older adults, we used the Montreal Cognitive Assessment (MoCA; [33]) to assess multiple cognitive domains, including attention, memory, language, and visuospatial skills. The mean MoCA score indicated that the older participants were cognitively well-preserved and none of their scores fell below the dementia cutoff (i.e., MoCA < 17, [34]).

To assess CR, the Cognitive Reserve Index Questionnaire (CRIq; [35]) was administered. This instrument estimates an individual’s CR on the basis of lifelong experiences, following the theoretical framework proposed by Stern [22]. The questionnaire comprises three domains: education, which assesses years of formal schooling; working activity, which considers both the type and duration of paid employment (with at least one year per job); and leisure time, which evaluates cognitively stimulating activities carried out outside of work or education, such as driving, managing finances, or attending cultural events, and categorizes them according to frequency (weekly, monthly, yearly, or fixed schedule). A total CRIq score is then derived from the combination of these three domains.

The EIS [27], a 33-item self-report questionnaire, was used to measure EI. The instrument assesses the individual’s ability to perceive, understand, manage and use emotions effectively, including skills such as emotional regulation and the strategic use of emotions in thinking and behaviour. Participants respond by indicating their degree of agreement with each statement on a 5-point Likert scale (from 1 = “Strongly disagree” to 5 = “Strongly agree”). The total score was obtained by summing the values of all items, ranging from 33 to 165, where higher scores indicate higher levels of EI (see Table 1 for scoring).

#### 2.2.2. Stimuli

The stimuli for the 1- and 2-back tasks consisted of 55 images of faces selected from the FACES database [36], a validated set of color photographs depicting frontal, naturalistic faces belonging to different age groups. The stimuli were the same as those used by Berger et al. [9], selected from a larger pool of 234 faces based on a preliminary rating study in which younger and older adults evaluated each face on valence and arousal. Faces with the highest agreement between age groups were retained, resulting in a set balanced for gender, age group of the face model, and emotional expression (happy, angry, neutral), with each picture depicting a unique individual. Both tasks were preceded by 12 practice trials.

#### 2.2.3. 1-Back Task

**Figure 1:**
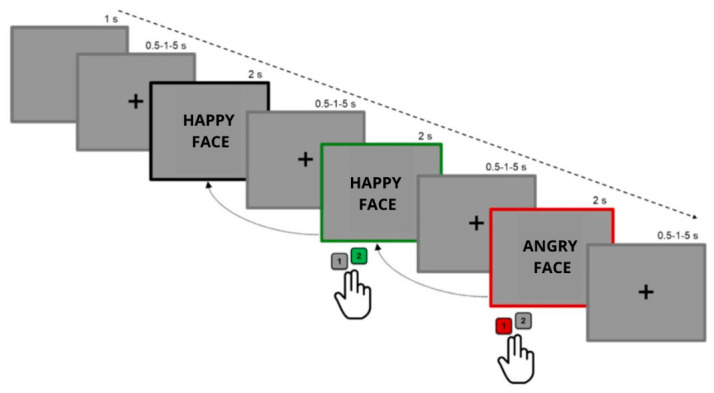
Example sequence of the 1-back Emo task. Each trial consisted of a face stimulus preceded by a fixation cross. Participants indicated whether the emotional expression of the current face matched that of the immediately preceding face. The green frame illustrates a correct response, whereas the red frame illustrates an incorrect response. Numbers below the stimuli indicate the response options (keys “F” and “K”). Timing information for stimulus presentation and inter-stimulus intervals is shown schematically above the sequence.

The 1-back task consisted of 165 trials divided into three blocks of 55 trials each. The order of the Emo and Age tasks was counterbalanced across participants. In the Emo task, participants were instructed to judge whether the emotional expression of the current face (happy, neutral or angry) matched that of the immediately preceding face. In the Age task, participants were instructed to judge whether the age category of the current face (young, middle-aged, or old) matched that of the immediately preceding face. The two tasks were identical in stimulus presentation and timing, differing only in the judgment dimension specified in the instructions (see Figure 1). At the beginning of each block, participants were instructed to observe the first face without pressing any key. Starting from the second face, they were required to indicate whether the current face matched or did not match the previous one by pressing the “F” or “K” key on the keyboard (the association between keys and match/no-match responses was counterbalanced across participants), resulting in 54 analysable trials per block. Each block included 18 trials for each age condition (i.e., young, middle-aged, and old), with 6 trials for each emotional condition (positive, neutral, and negative). Within each emotional condition, 3 trials depicted male faces and 3 depicted female faces.

#### 2.2.4. 2-Back Task

The 2-back task consisted of 190 trials divided into five blocks of 38 items each. This structure followed the paradigm established by Berger et al. [9], in which the first two trials of each block are non-analysable, unlike the 1-back task where only the first trial is excluded. This procedure allowed the number of analyzable trials to remain comparable across difficulty conditions.

**Figure 2:**
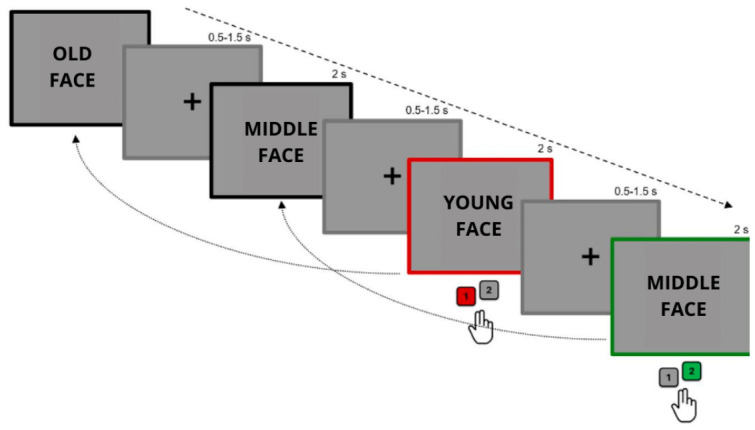
Example sequence of the 2-back Age task. The figure illustrates a schematic sequence of face stimuli used in the 2-back Age task. Participants judged whether the age of the current face matched that of the face presented two trials earlier. A green frame indicates a correct response, whereas a red frame indicates an incorrect response. Numbers below the stimuli represent the response options (keys “F” and “K”), and stimulus timing is schematically depicted above the sequence.

As in the 1-back task, the order of the Age and Emo tasks and the assignment of match/no-match responses to the response keys were counterbalanced across participants. However, participants were required to compare the current face with the one presented two trials earlier. In the Emo task, participants judged whether the emotional expression matched that of the face presented two trials earlier, whereas in the Age task, they judged whether the age category matched (see Figure 2). Starting from the third stimulus, participants responded by pressing the “F” or “K” key on the keyboard, resulting in 36 analyzable trials per block. Each block included 12 trials for each age condition (young, middle-aged, and old), with 4 trials for each emotional condition (positive, neutral, and negative). Within each emotional condition, two trials depicted male faces and two depicted female faces.

### 2.3. Data Analysis

All statistical analyses were performed in R v.4.2.2 [37]. We used as dependent variables both accuracy and reaction times (RT). Given that the normality assumption for traditional ANOVA was not met for both measures (Shapiro–Wilk test, *p* < 0.05), mixed-effects models were employed. Accuracy was coded as a binary variable (0 = incorrect, 1 = correct). To account for the binary nature of the dependent variable, generalized linear mixed-effects models (GLMMs) with a binomial distribution and logit link function were fitted. Two models were estimated: the first included only the within-participants experimental factors, whereas the second additionally included Cognitive Reserve (CR) and Emotional Intelligence (EI) as continuous covariates. The fixed factors included Emotion (happy, neutral, angry), Difficulty (1-back vs. 2-back), and Task (Emo vs. Age). All models included random intercepts for participants. Random slopes were not included because of convergence and model-complexity issues. Type III Wald chi-square tests were used to assess the significance of fixed effects. For all the analyses, the significance threshold was set at *p* < 0.05. Post hoc comparisons were performed on estimated marginal means (EMMs) using the *emmeans* package (version 2.0.3) [38], and Bonferroni correction applied.

## 3. Results

### 3.1. Accuracy

Accuracy results revealed a significant main effect of Task, χ^2^(1) = 24.85, *p* < 0.001, with higher accuracy in the Emo task than in the Age task. The main effect of Emotion was not significant (χ^2^(2) = 1.22, *p* = 0.544). Accuracy was also significantly lower in the 2-back compared to the 1-back condition, χ^2^(1) = 170.81, *p* < 0.001. Raw descriptive statistics for accuracy across Task, Emotion, and Difficulty conditions are reported in Table 2.

A significant interaction emerged between Task and Difficulty (χ^2^(1) = 21.13, *p* < 0.001). Post hoc comparisons revealed that, in the 1-back condition, participants showed higher accuracy in the Emo task than in the Age task (*b* = –0.583, *SE* = 0.045, *z* = –13.02, *p* < 0.0001). In contrast, in the 2-back condition, the difference between the Emo and Age tasks was not significant (*b* = 0.027, *SE* = 0.035, *z* = 0.76, *p* = 0.445).

Moreover, accuracy was significantly higher in the 1-back condition than in the 2-back condition, both in the Age task (*b* = 0.803, *SE* = 0.038, *z* = 20.95, *p* < 0.0001) and in the Emo task (*b* = 1.412, *SE* = 0.043, *z* = 33.08, *p* < 0.0001), reflecting the expected impact of cognitive load on performance.

A significant interaction between Task and Emotion also emerged (χ^2^(2) = 12.34, *p* = 0.002). Post hoc comparisons showed that, in the angry condition, participants were significantly more accurate in the Emo task than in the Age task (*b* = −0.150, *SE* = 0.047, *z* = −3.17, *p* = 0.0015). This difference was even larger for happy faces (*b* = −0.432, *SE* = 0.050, *z* = −8.57, *p* < 0.0001) and remained significant for neutral expressions (*b* = −0.253, *SE* = 0.049, *z* = −5.16, *p* < 0.0001), indicating overall higher accuracy in the emotion discrimination task across all emotional conditions.

Within-task comparisons further revealed that, in the Emo task, accuracy was significantly higher for happy faces than for angry faces (*b* = −0.36, *SE* = 0.051, *z* = −7.09, *p* < 0.0001) and neutral expressions (*b* = 0.20, *SE* = 0.053, *z* = 3.87, *p* = 0.0003). No significant differences between emotional conditions emerged in the Age task.

A significant three-way interaction between Task, Emotion, and Difficulty was observed for accuracy (χ^2^(2) = 8.71, *p* = 0.013). Specifically, in the 1-back condition, accuracy in the Emo task was significantly higher than in the Age task for angry faces (*b* = −0.369, *SE* = 0.0736, *z* = −5.01, *p* < 0.0001), happy faces (*b* = −0.718, *SE* = 0.0808, *z* = −8.88, *p* < 0.0001), and neutral faces (*b* = −0.674, *SE* = 0.0783, *z* = −8.61, *p* < 0.0001).

Within-task contrasts revealed no significant emotion-related differences in the Age task. In contrast, in the Emo task, accuracy was significantly higher for happy than angry faces (*b* = −0.416, *SE* = 0.0833, *z* = −4.99, *p* < 0.0001), and significantly higher for neutral than angry faces (*b* = −0.302, *SE* = 0.0812, *z* = −3.72, *p* = 0.0006). No significant differences emerged between happy and neutral faces.

In the 2-back condition, task effects varied as a function of Emotion. No significant difference emerged between the Age and Emo tasks for angry faces (*b* = 0.049, *SE* = 0.059, *z* = 0.805, *p* = 0.4206). However, for happy faces, accuracy was significantly higher in the Emo task than in the Age task (*b* = −0.175, *SE* = 0.0611, *z* = −2.86, *p* = 0.0042), whereas for neutral faces, performance was significantly better in the Age task than in the Emo task (*b* = 0.145, *SE* = 0.0602, *z* = 2.41, *p* = 0.0162).

Within-task comparisons in the 2-back condition again revealed no significant emotion-related differences in the Age task, whereas in the Emo task, happy faces elicited significantly higher accuracy than both angry (*b* = −0.320, *SE* = 0.0608, *z* = −5.26, *p* < 0.0001) and neutral faces (*b* = 0.301, *SE* = 0.0604, *z* = 4.98, *p* < 0.0001). No significant differences emerged between happy and neutral faces.

**Figure 3:**
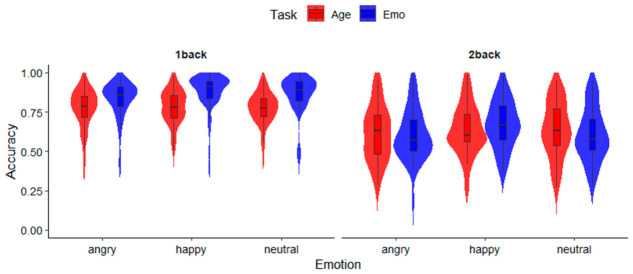
Violin plots illustrating accuracy (proportion correct) across Task, Emotion, and Difficulty conditions. Embedded boxplots represent the interquartile range and median. Emotion-related accuracy effects are amplified under high cognitive load (2-back), particularly in the Emo task.

Together, these findings indicate that the interaction between task type and emotional expression in predicting accuracy was modulated by task difficulty, with emotional effects particularly pronounced in the Emo task under higher cognitive load (2-back) (see Figure 3).

The full set of main effects and interactions from the GLMM, including those not described in detail above, is reported in Table 3.

### 3.2. Reaction Time

RT analyses revealed a significant main effect of Task (χ^2^(1) = 4.26, *p* = 0.039), with RTs being slightly slower in the Emo task than in the Age task (*b* = −0.023, *SE* = 0.011, *t* = −2.07, *p* = 0.039). The main effect of Emotion was also significant (χ^2^(2) = 6.21, *p* = 0.045). Pairwise comparisons indicated a graded effect of emotional expression, with RTs being fastest for happy faces, intermediate for neutral faces, and slowest for angry faces. Specifically, RTs were significantly faster for happy than neutral (*p* = 0.035) and angry (*p* < 0.001) faces, and for neutral than angry faces (b = −0.026, SE = 0.011, t = −2.30, *p* = 0.022). Finally, RT analyses revealed a significant main effect of Difficulty (χ^2^(1) = 14.92, *p* < 0.001), with slower RTs in the 2-back than in the 1-back condition (*b* = 0.051, *SE* = 0.013, *t* = 3.86, *p* < 0.001) Raw descriptive statistics for reaction times across Task, Emotion, and Difficulty conditions are reported in Table 4.

**Figure 4:**
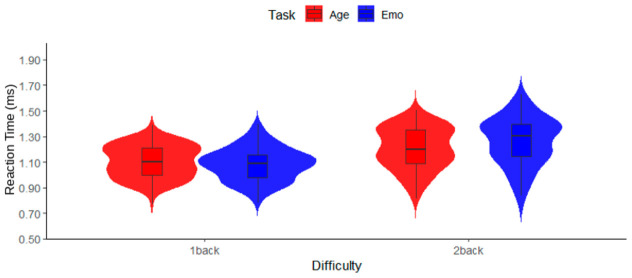
Violin plots illustrating reaction times (RTs, in ms) as a function of Task (Age vs. Emo) and Difficulty (1-back vs. 2-back). Embedded boxplots represent the interquartile range and median. The Task × Difficulty interaction shows an inversion of task-related effects across cognitive load, with larger task differences emerging in the 2-back condition.

A significant interaction was observed between Difficulty and Task (χ^2^(1) = 33.41, *p* < 0.001). Post hoc comparisons revealed that in the 1-back condition, participants responded significantly slower during the Age task compared to the Emotion task (*b* = 0.027, *SE* = 0.006, *z* = 4.25, *p* < 0.0001). Conversely, in the 2-back condition, the effect reversed: participants were significantly slower in the Emotion task than in the Age task (*b* = −0.053, *SE* = 0.008, *z* = −6.22, *p* < 0.0001) (see Figure 4).

A further significant interaction was found between Task and Emotion, with χ^2^(2) = 11.95, *p* < 0.001.

**Figure 5:**
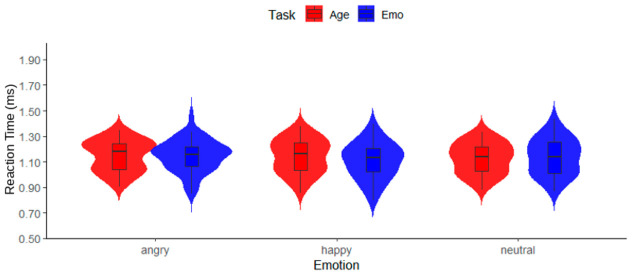
Violin plots illustrating RTs (in ms) as a function of Task (Age vs. Emo) and Emotion (angry, happy, neutral). Embedded boxplots represent the interquartile range and median. Task-related differences in RTs vary across emotional conditions, with slower responses in the Emo task for angry and neutral stimuli, and comparable performance between tasks for happy faces.

Post hoc comparisons revealed that when angry stimuli were presented, participants responded significantly faster in the Age task compared to the Emo task (*b* = −0.031, *SE* = 0.009, *z* = −3.35, *p* = 0.0008). A similar pattern was observed for neutral stimuli, with faster responses in the Age task relative to the Emo task (*b* = −0.022, *SE* = 0.009, *z* = −2.40, *p* = 0.016). In contrast, no significant difference between tasks emerged for happy stimuli (*b* = −0.013, *SE* = 0.009, *z* = 1.53, *p* = 0.12) (see Figure 5).

Examining emotional effects within each task revealed no significant differences among emotions in the Age task. However, in the Emo task, responses to angry stimuli were significantly slower than responses to happy stimuli (*b* = 0.052, *SE* = 0.009, *z* = 5.78, *p* < 0.0001), while responses to happy faces were faster than to neutral stimuli (*b* = −0.032, *SE* = 0.009, *z* = −3.56, *p* = 0.001). Finally, the difference between angry and neutral faces was not significant (*b* = −0.020, *SE* = 0.009, *z* = 2.19, *p* = 0.084). The full set of main effects and interactions from the RT model, including those not described in detail above, is reported in Table 5.

### 3.3. Cognitive Reserve and Emotional Intelligence

To examine the role of individual differences, two separate mixed-effects models were estimated, one including CR and one including EI as continuous predictors, each entered in interaction with the experimental factors Task, Emotion, and Difficulty.

#### 3.3.1. Accuracy and CR

**Figure 6:**
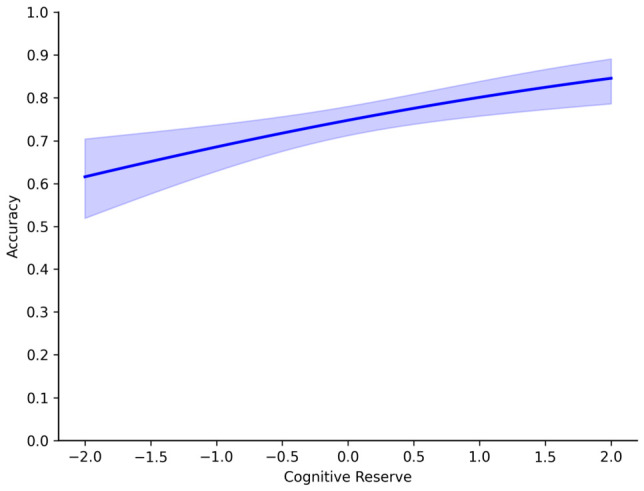
Model-predicted accuracy as a function of CRI, averaged across Task, Emotion, and Difficulty. The blue line represents the estimated marginal effect of CR, while the shaded area indicates the 95% confidence interval. The *x*-axis represents CRI values standardised as z-scores (range: −2 to +2); the *y*-axis represents predicted accuracy (proportion correct, range: approximately 0.60 to 0.90).

The analysis revealed a significant main effect of CR on accuracy (χ^2^(1) = 8.30, *p* = 0.004), indicating that higher CR was associated with greater accuracy across conditions (see Figure 6). This effect was further supported by the overall estimated marginal slope of CRI_z, averaged across Task, Emotion, and Difficulty (*b* = 0.307, *SE* = 0.09, *z* = 3.41, *p* = 0.0006), confirming a positive association between CR and accuracy. No significant interactions emerged between CRI and other variables.

#### 3.3.2. Accuracy and EI

In contrast to CRI, no main effect of EI on accuracy was observed (χ^2^(1) = 0.06, *p* = 0.803), indicating that overall accuracy was not directly associated with individual differences in emotional intelligence. No significant interactions emerged between EI and other variables.

#### 3.3.3. RT and CR

The analysis revealed no main effect of CR on RT (χ^2^(1) = 0.003, *p* = 0.957), indicating that overall reaction times were not directly associated with cognitive reserve. Consistently, the estimated fixed effect of CRI on RT was not significant (*b* = −0.00007, *SE* = 0.00122, t = −0.05).

**Figure 7:**
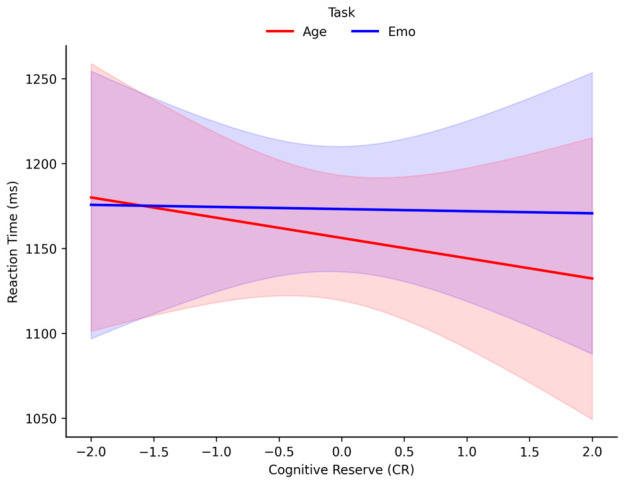
RTs as a function of CR for the Age and Emo tasks. Lines represent model-estimated RTs from a linear mixed-effects model that includes CR as a continuous predictor, with Task, Emotion, and Difficulty as fixed factors and random intercepts for participants. The *x*-axis represents CRI values standardized as z-scores (range −2 to +2); the *y*-axis represents predicted RTs in milliseconds. Shaded areas indicate 95% confidence intervals. The Task × CR interaction was significant, reflecting a numerically negative slope in the Age task and a near-zero slope in the Emo task; neither simple slope was individually significant.

However, a significant interaction between Task and CR emerged (χ^2^(1) = 4.89, *p* = 0.027), indicating that the association between CR and RT differed between tasks. Follow-up analyses of the simple slopes, however, indicated that this association was not reliably different from zero in either task considered separately: in the Emo task (M = 1.17, SE = 0.02; b = 0.0005, SE = 0.00115, t = 0.43, *p* = 0.665); and in the Age task (M = 1.16, SE = 0.02; b = −0.00052, SE = 0.00115, t = −0.46, *p* = 0.645). The significant Task × CR interaction therefore reflects a genuine difference between the two slopes (one numerically negative, one numerically positive) rather than a reliable effect of CR on RT within either task individually. No other interactions involving CR reached significance (see Figure 7).

#### 3.3.4. RT and EI

**Figure 8:**
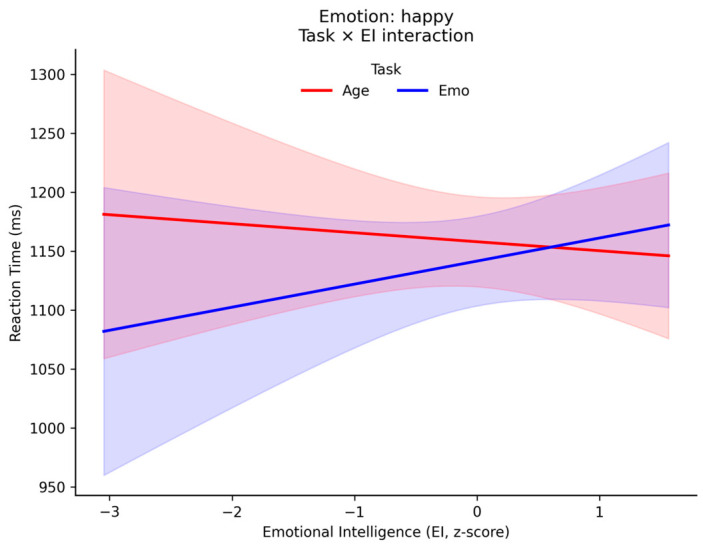
Task × EI interaction on RTs for Emotion happy. Model-estimated RTs are plotted as a function of EI separately for the Age and Emo Tasks. The *x*-axis represents EI raw scores; the *y*-axis represents predicted RTs in milliseconds. Shaded bands indicate 95% confidence intervals. Post hoc analyses revealed opposite associations between EI and RT across tasks, with higher EI predicting faster responses in the Age task and slower responses in the Emo task for happy faces.

The analysis revealed no main effect of EI on RT (χ^2^(1) = 0.31, *p* = 0.576), indicating that RTs were not directly related to individual differences in EI. In addition, a significant interaction between Task, Emotion, and EI emerged (χ^2^(2) = 7.20, *p* = 0.027). Follow-up analyses showed a significant task-related difference in the association between EI and RTs for happy faces only (*b* = −0.00152, *SE* = 0.00061, *t* = −2.50, *p* = 0.012), such that higher EI was associated with faster RTs in the Age task (*M* = 1.16, *SE* = 0.02) and slower RTs in the Emo task (*M* = 1.17, *SE* = 0.02). We note that the present study did not directly or indirectly assess the cognitive or attentional processes underlying these reaction time differences. Accordingly, the observed association should be interpreted as a descriptive pattern rather than as evidence for a specific underlying mechanism, such as differences in response strategy. No task-related differences in the association between EI and RTs were observed for angry (*p* = 0.355) or neutral faces (*p* = 0.789). No other main effects or interactions involving EI reached significance (see Figure 8).

Taken together, this pattern indicates that the association between EI and reaction times was not uniform across emotional conditions, but was instead confined to happy expressions, for which it showed opposite directions depending on whether the emotional content was task-relevant or task-irrelevant. Specifically, in the Age task, where facial emotion was not directly relevant to the response, higher EI was associated with progressively faster responses to happy faces. Conversely, in the Emo task, where participants explicitly judged the emotional expression, higher EI was associated with progressively slower responses to the same happy faces. No comparable task-dependent pattern emerged for angry or neutral expressions, for which the association between EI and RTs did not differ between the Age and Emo tasks. This three-way interaction therefore indicates that the association between EI and reaction times depends jointly on the relevance of the emotional content to the task and the emotional valence, rather than reflecting a generalized facilitation or slowing of responses.

## 4. Discussion

The present study investigated how CR and EI relate to working memory performance in older adults under varying levels of cognitive load and emotional relevance using an emotional variant of the n-back task. Overall, the findings confirmed robust load-related performance costs and demonstrated that emotional modulation of working memory is highly context-dependent, emerging selectively when emotional information is task-relevant and cognitive demands are elevated.

As expected, increasing task difficulty was associated with reduced accuracy and slower reaction times, consistent with well-established evidence on age-related limitations in working memory updating and interference control [1,7]. Beyond load effects, task relevance critically shaped performance patterns. Emotional stimuli modulated both accuracy and reaction times selectively in the Emo task, whereas no reliable emotional effects emerged when emotional expressions were irrelevant to task goals (Age task). This dissociation supports the dual competition model [15], according to which emotional information influences performance only when it competes for, or is allocated, shared cognitive resources.

Emotional valence further modulated performance in a selective manner. Happy facial expressions were associated with faster responses and higher accuracy than angry expressions, particularly when emotions were task-relevant. This pattern is consistent with the motivational priorities described by socioemotional selectivity theory, according to which older adults preferentially orient toward positive and meaningful information as a function of perceived time horizons [20,21], and suggests that emotional facilitation reflects a goal-directed bias rather than an automatic processing advantage.

Importantly, however, happy and angry faces differ not only in valence but also in arousal, with angry faces generally perceived as more arousing. This difference may have contributed to the observed effects, given that arousal can independently modulate emotional processing in older adults [39]. Notably, emotional effects on accuracy were amplified under high cognitive load, suggesting that limited cognitive resources increase the functional impact of affective salience. Within a dynamic gating framework [3], these effects may reflect a more challenging balance between flexibility and interference control when both cognitive and emotional demands are elevated.

Regarding individual differences, CR was associated with higher accuracy across conditions, independently of task type, emotional content, or cognitive load. This finding supports the view that CR primarily contributes to representational stability and executive efficiency rather than to context-specific emotional modulation [24,40]. Importantly, CR did not interact with task demands or emotional factors, suggesting a domain-general contribution to performance stability rather than a context-dependent compensatory mechanism.

In contrast, EI was not associated with overall accuracy and showed no main effect on reaction times. However, EI selectively modulated response speed as a function of task relevance and emotional content. Specifically, higher EI was associated with faster responses for happy faces in the Age task but with slower responses in the Emo task. This pattern suggests that self-reported EI is associated with different response speed profiles depending on the functional relevance of emotional information, rather than uniformly enhancing or impairing processing speed. We note that the present study did not assess the cognitive or attentional processes underlying these reaction time effects; consequently, although differential resource allocation or more extensive processing of affective content offer plausible explanations, these interpretations remain speculative and cannot be directly inferred from the present data.

Some caution is warranted in interpreting the emotional valence effects, as happy and angry faces may differ not only in emotional valence but also in perceptual salience and arousal. In addition, the relatively small sample size may limit the generalizability of the individual-difference effects involving CR and EI. A further limitation of this study is that the present analyses focused exclusively on the older adult sample. Although data from a younger adult comparison group were also collected as part of the broader research project from which the present sample was drawn, those data were not included in the present analyses, which focused specifically on individual differences in CR and EI within older adulthood. As a result, the present design does not allow us to determine whether the observed emotion–cognition interactions and individual-difference effects are specific to older adulthood or are also present earlier in adulthood. Future work directly comparing younger and older adults within the same analytic framework would help clarify the developmental specificity of these effects.

Beyond the limitations already noted, the present study has several strengths worth highlighting. The use of a within-subjects factorial design crossing task relevance, emotional valence, and cognitive load enabled direct comparisons of emotional modulation under task-relevant and task-irrelevant conditions within the same participants, increasing statistical power and reducing between-subjects confounds. The inclusion of two theoretically grounded individual-difference measures, CR and EI, within a single sample also enabled us to examine their dissociable contributions as cognitive and emotional resources, rather than inferring this distinction from separate studies or samples.

These considerations suggest several specific avenues for future research aimed at clarifying the mechanisms linking CR, EI, and working memory performance in ageing. Longitudinal designs would help establish whether CR and EI predict individual trajectories of change in emotion–cognition interactions over time, rather than merely reflecting cross-sectional differences. Studies incorporating neuroimaging or psychophysiological measures could help identify the neural and autonomic correlates of the dissociable contributions of CR and EI identified here, for example by testing whether CR relates to more efficient recruitment of executive control networks, whereas EI relates to differential engagement of affective processing regions during task-relevant versus task-irrelevant emotional processing. Finally, future studies directly assessing emotion regulation strategies, using self-report measures of habitual regulation style, experience-sampling approaches, or behavioral indices of attentional engagement with emotional stimuli, would allow the speculative interpretations advanced in this discussion regarding the strategic role of EI to be tested directly, rather than inferred from reaction time patterns alone.

Together, these findings indicate that CR and EI contribute to working memory performance through partially dissociable mechanisms. CR appears to support accuracy consistently across conditions, whereas EI primarily modulates response dynamics in emotionally salient contexts without directly improving accuracy. Overall, these findings suggest that successful cognitive functioning in ageing depends not only on preserved executive resources, but also on the efficient regulation of emotionally salient information.

## 5. Conclusions

In conclusion, the present study shows that emotional modulation of working memory performance in ageing is highly context-dependent, emerging primarily when emotional information is task-relevant and cognitive demands are elevated. Cognitive reserve and emotional intelligence contributed to working memory performance through distinct mechanisms: cognitive reserve supported overall accuracy across conditions, whereas emotional intelligence selectively shaped response dynamics in emotionally salient contexts without conferring a general performance advantage.

These dissociable contributions highlight the importance of integrative models of cognitive ageing that account not only for cognitive resources, but also for individual differences in affective processing and regulation. Considering both domains may provide a more comprehensive understanding of variability in executive functioning in later life and of the mechanisms supporting adaptive cognitive functioning in ageing.

## Figures and Tables

**Table 1:** Descriptive Statistics of Socio-demographic and Cognitive Measures.

Index	Mean (SD)
Age	69.83 (5.90)
Education (years)	14.20 (3.96)
MoCA	26.62 (3.03)
Cognitive Reserve (CRIq)	128.24 (18.65)
Emotional Intelligence Scale (EIS)	133.08 (14.83)

**Table 2:** Raw mean accuracy (proportion correct) and SD as a function of Task, Emotion, and Difficulty.

Task	Emotion	Difficulty	Raw M	SD
Age	Angry	1-back	0.77	0.13
Emo	Angry	1-back	0.83	0.12
Age	Happy	1-back	0.78	0.11
Emo	Happy	1-back	0.88	0.12
Age	Neutral	1-back	0.77	0.10
Emo	Neutral	1-back	0.86	0.12
Age	Angry	2-back	0.61	0.17
Emo	Angry	2-back	0.60	0.16
Age	Happy	2-back	0.63	0.16
Emo	Happy	2-back	0.67	0.15
Age	Neutral	2-back	0.64	0.18
Emo	Neutral	2-back	0.61	0.17

**Table 3:** Main effects and interactions for Accuracy.

Effect	χ^2^	df	*p*-Value
Intercept	136.9	1	<0.001
Task	24.85	1	<0.001
Emotion	1.22	2	0.544
Difficulty	170.81	1	<0.001
Task × Emotion	12.34	2	0.002
Task × Difficulty	21.13	1	<0.001
Emotion × Difficulty	1.69	2	0.430
Task × Emotion × Difficulty	8.71	2	0.013

**Table 4:** Raw mean reaction times (RTs, in milliseconds) and SD as a function of Task, Emotion, and Difficulty.

Task	Emotion	Difficulty	Raw M (ms)	SD
Age	Angry	1-back	1112.1	122.3
Emo	Angry	1-back	1095.7	117.5
Age	Happy	1-back	1111.7	126.7
Emo	Happy	1-back	1059.9	134.0
Age	Neutral	1-back	1088.0	136.0
Emo	Neutral	1-back	1090.1	129.6
Age	Angry	2-back	1227.7	168.5
Emo	Angry	2-back	1281.2	177.8
Age	Happy	2-back	1200.7	170.9
Emo	Happy	2-back	1213.0	191.7
Age	Neutral	2-back	1186.5	151.8
Emo	Neutral	2-back	1281.7	195.0

**Table 5:** Main effects and interactions for Reaction Time (RT).

Effect	χ^2^	df	*p*-Value
Intercept	2975.13	1	<0.001
Task	4.26	1	0.039
Emotion	6.21	2	0.045
Difficulty	14.92	1	<0.001
Task × Emotion	11.95	2	0.003
Task × Difficulty	33.41	1	<0.001
Emotion × Difficulty	4.51	2	0.105
Task × Emotion × Difficulty	4.99	2	0.082

## Data Availability

The data presented in this study are available on request from the corresponding author. The data are not publicly available due to ethical restrictions.
